# Supplementary material for: Enantioselective Binding of Proton Pump Inhibitors to Alpha1-Acid Glycoprotein and Human Serum Albumin—A Chromatographic, Spectroscopic, and In Silico Study
Source: Int J Mol Sci. 2024 Oct 1;25(19):10575. doi: 10.3390/ijms251910575 (PMC11477000; doi:10.3390/ijms251910575)
Supplement: Supplementary file 1 [file ijms-25-10575-s001.zip › ijms-3184177-supplementary.pdf]

Supplementary Information for

Enantioselective binding of proton pump inhibitors to alpha1-acid glycoprotein and human serum albumin - A chromatographic, spectroscopic and in silico study

Gergely Dombi[1], Levente Tyukodi[2], Máté Dobó[1], Gergely Molnár[1], Zsuzsanna Rozmer[2], Zoltán-István Szabó[3,4], Béla Fiser[5,6,7] and Gergő Tóth[1*]

[1] Department of Pharmaceutical Chemistry, Semmelweis University, Hogyes E. 9, H-1092 Budapest, Hungary
[2] Department of Pharmaceutical Chemistry, University of Pécs, H-7624 Pécs, Hungary
[3] Department of Pharmaceutical Industry and Management, Faculty of Pharmacy, George Emil Palade University of Medicine, Pharmacy, Science, and Technology of Targu Mures, Gh. Marinescu 38, 540142 Targu Mures, Romania
[4] Sz-imfidum Ltd., 525401 Lunga nr 504, 525401 Targu Mures, Romania
[5] Institute of Chemistry, University of Miskolc, Miskolc-Egyetemváros 3515, Hungary
[6] Department of Biology and Chemistry, Ferenc Rakoczi II Transcarpathian Hungarian College of Higher Education, Transcarpathia, 90200 Beregszasz, Ukraine
[7] Department of Physical Chemistry, Faculty of Chemistry, University of Lodz, 90-149 Łódź, Poland
[*] Correspondence: toth.gergo@semmelweis.hu

Table of Contents

Supplementary Table 1. Comparison of recent study results with previous literature

Supplementary Figure 1. Chromatograms obtained using different buffer constituents on HAS column: A – 10 mM sodium phosphate, pH 7; B – 10 mM ammonium acetate, pH 7; C – 10 mM ammonium acetate, pH 7; D – 10 mM sodium citrate, pH 7. The column temperature is 25 °C, and the applied organic modifier is 1% 2-propanol

Supplementary Table 2. Retention times and resolution values using different buffer constituents on the AGP and HSA columns at 25 °C. The applied organic modifier is 2.5% 2-propanol for the AGP column and 1% 2-propanol for the HSA column

Supplementary Figure 2. Representative spectra obtained from fluorescence measurements: A – Unprocessed fluorescence spectra of R-lansoprazole and AGP; B – Unprocessed fluorescence spectra of S-lansoprazole and AGP; C – Emission spectra of the investigated compounds; D – Background fluorescence of the matrix (phosphate buffer).

Supplementary Table 3. Absorbance values used for the inner filter effect calculation for lansoprazole. The absorbance values for the specified solution compositions were measured with UV-Vis spectrophotometer as part of fluorescence quenching studies, with an excitation wavelength of 295 nm and an emission wavelength of 340 nm.

Supplementary Table 4. The observed and IFE corrected fluorescence intensity values for S-lansoprazole and R-lansoprazole, respectively.

Supplementary Figure 3. Stern-Volmer plots for S-lansoprazole and R-lansoprazole. Although Stern-Volmer plots typically exhibit an increasing trend, the scatter plots here show minimal linearity in the data points (r^2 = 0.3371; r^2 = 0.4126).

Supplementary Figure 4. Representative UV spectra: A – R-lansoprazole and AGP (1 μM); B – S-lansoprazole and AGP (1 μM); C – R-rabeprazole and AGP (1 μM); D – S-rabeprazole and AGP (1 μM)

Supplementary Figure 5. Examples of fitting the Benesi-Hildebrandt equation. The r^2 value is higher than 0.995 in all cases: A – S-rabeprazole (r^2 = 0.9994); B – R-omeprazole (r^2 = 0.9979); C – S-omeprazole (r^2 = 0.9979).

Supplementary Table 5. Changes in retention factor values in the presence of warfarin and ibuprofen at different concentrations on HSA column. (Chromatographic parameter: Column temperature: 25 °C, mobile phase: 2-propanol : 10 mM pH 7 sodium phosphate buffer 1:99, flow rate: 0.7 mL/min, detection wavelength: 210 nm).

Supplementary Figure 6. The fluorescence spectra of HSA-warfarin complex titrated with S-omeprazole

Supplementary Figure 7. The potential binding sites in HSA identified by Sitemap

Supplementary Table 6. Docking scores of PPIs at potential binding sites of HSA

Supplementary Table 1. Comparison of recent study results with previous literature

Investigated compounds	Protein	Enantio-selectivity	Method(s)	Main result(s)	Ref.
Omeprazole, Pantoprazole, Ilaprazole	BSA	NO	fluorescence, UV–vis circular dichroism (CD)	Omeprazole (log*K*=4.58)<br>Pantoprazole (log*K*=5.04)<br>Ilaprazole (log*K*=5.63)<br>Binding to subdomain IIA	[37]
Omeprazole	BSA	NO	Fluorescence	K=0.068 (μM^{-1})	[38]
Omeprazole and S-omeprazole	HSA	partially*	Fluorescence, CD, voltametry and in silico	Omeprazole (log*K*=4.61)<br>*S*-Omeprazole (log*K*=4.70)<br>Binding to subdomain IIA	[39]
R-lansoprazole	HSA	partially**	Fluorescent, UV and molecular docking	log*K*=3.44<br>Bind to subdomain IIA	[40]
S-Omeprazole<br>R-Omeprazole	HSA	yes	Affinity capillary electrophoresis, fluorescence	R-omeprazole (log*K*=3.50)<br>S-omeprazole (log*K*=3.73)<br>Enantioselective binding<br>S-omeprazole bind to subdomain IIA,<br>R-omeprazole bind to subdomain IIIA	[41]
Omeprazole and its metabolite	AGP	yes	HPLC using AGP column	Stereoselective binding	[42]
S-Omeprazole,<br>R-omeprazole<br>S-lansoprazole<br>R-lansoprazole<br>S-rabeprazole<br>R-rabeprazole	HSA,<br>AGP	yes	HPLC, fluorescence, UV, in silico	Stereoselective binding<br>S-omeprazole - HSA (b% 91.76, logK=4.02)<br>R-omeprazole – HSA (b% 91.76, logK=4.47)<br>S-lansoprazole - HSA (b% 94.64, logK=4.66)<br>R-lansoprazole- HSA (b% 92.45, logK=4.22)<br>S-rabeprazole - HSA (b% 88.84, logK=4.42)<br>R-rabeprazole - HSA (b% 88.84, logK=4.33)<br>S-omeprazole - AGP (b% 97.23, logK=4.47)<br>R-omeprazole – AGP (b% 96.65, logK=4.69)<br>S-lansoprazole - AGP (b% 96.86, logK=4.83)<br>R-lansoprazole- AGP (b% 97.56, logK=4.56)<br>S-rabeprazole – AGP (b% 97.98, logK=4.76)<br>R-rabeprazole - AGP (b% 96.78, logK=4.50)<br>Binding to subdomain IIA and IIIA	This work

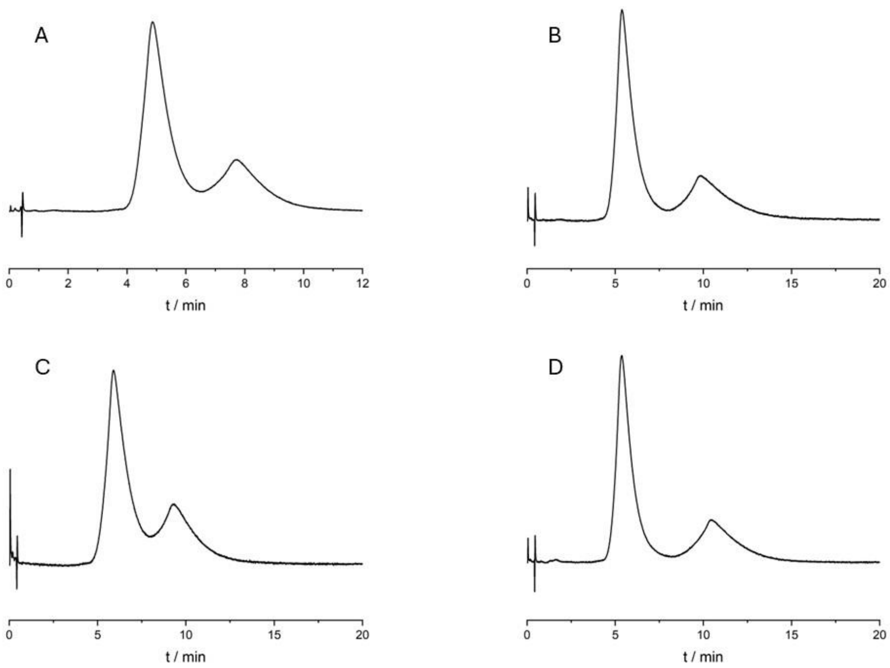

Supplementary Figure 1. Chromatograms obtained using different buffer constituents on HSA column: A – 10 mM sodium phosphate, pH 7; B – 10 mM ammonium acetate, pH 7; C – 10 mM ammonium acetate, pH 7; D – 10 mM sodium citrate, pH 7. The column temperature is 25 °C, and the applied organic modifier is 1% 2-propanol

Supplementary Table 2. Retention times and resolution values using different buffer constituents on the AGP and HSA columns at 25 °C. The applied organic modifier is 2.5% 2-propanol for the AGP column and 1% 2-propanol for the HSA column

		Omeprazole			Lansoprazole			Rabeprazole		
column	Buffer	t_1	t_2	R_s	t_1	t_2	R_s	t_1	t_2	R_s
AGP	phosphate	23.50	28.34	1.2	27.64	34.00	1.5	26.23	37.39	2.5
	Acetate	22.24	28.83	1.3	26.82	32.32	0.8	26.30	36.03	1.5
	Bicarbonate	21.01	23.89	0.61	22.79	35.16	1.7	24.27	30.84	0.81
	citrate	28.99	38.79	1.3	28.44	45.79	2.2	31.89	34.96	0.49
HSA	phosphate	4.00	4.00	-	4.87	7.71	1.8	3.12	3.12	-
	Acetate	5.90	5.90	-	5.39	9.85	1.8	3.66	3.66	-
	Bicarbonate	6.52	6.52	-	5.92	9.35	1.2	4.01	4.01	-
	citrate	6.13	6.13	-	5.37	10.46	1.9	3.57	3.57	-

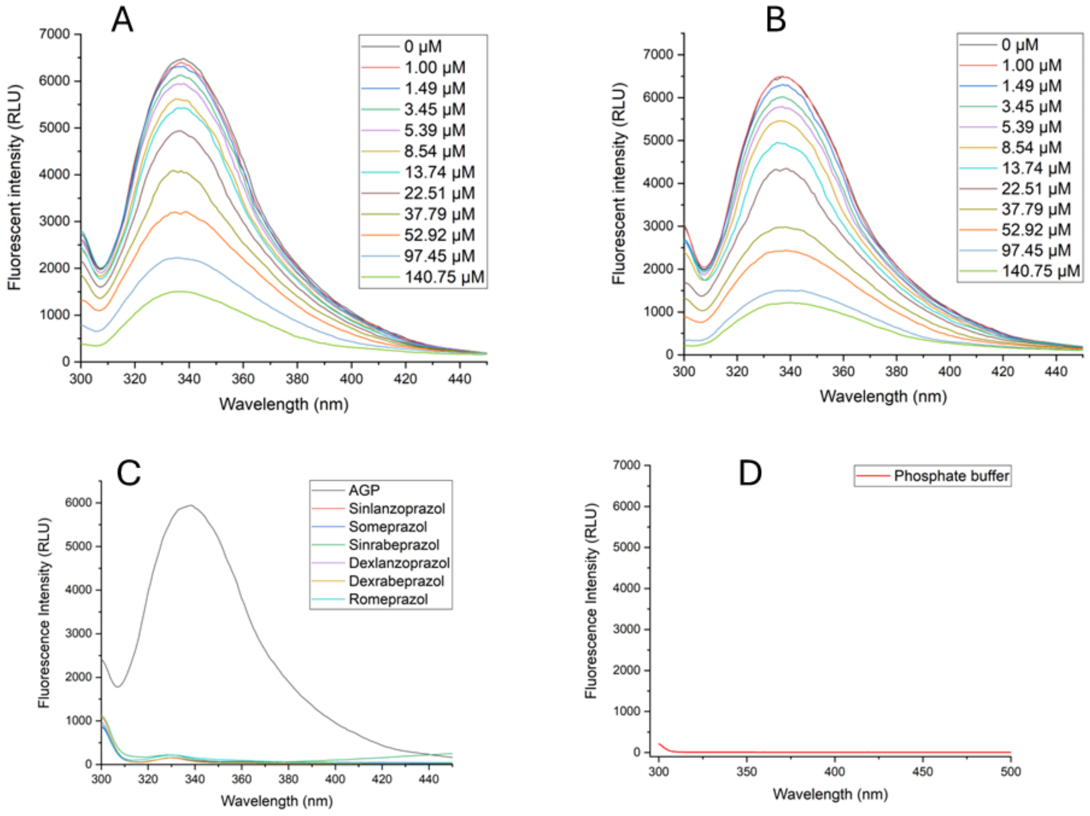

Supplementary Figure 2. Representative spectra obtained from fluorescence measurements: A – Unprocessed fluorescence spectra of R-lansoprazole and AGP; B – Unprocessed fluorescence spectra of S-lansoprazole and AGP; C – Emission spectra of the investigated compounds; D – Background fluorescence of the matrix (phosphate buffer).

Supplementary Table 3. Absorbance values used for the inner filter effect calculation for lansoprazole. The absorbance values for the specified solution compositions were measured with UV-Vis spectrophotometer as part of fluorescence quenching studies, with an excitation wavelength of 295 nm and an emission wavelength of 340 nm.

Concentration (μM)	S-Lansoprazole		R-Lansoprazole	
0.00	295 nm	338 nm	295 nm	338 nm
1.00	0.1375	0.0274	0.1319	0.0272
1.49	0.1496	0.0275	0.1426	0.0274
3.45	0.1981	0.0281	0.1856	0.0284
5.39	0.2461	0.0287	0.2282	0.0294
8.54	0.3238	0.0297	0.2971	0.0310
13.74	0.3169	0.0293	0.3989	0.0315
22.51	0.3266	0.0296	0.4779	0.0317
37.79	0.3434	0.0300	0.6154	0.0320
52.92	0.3600	0.0305	0.7516	0.0323
97.45	0.4090	0.0318	1.1524	0.0331
140.74	0.4566	0.0331	1.5420	0.0340

Supplementary Table 4. The observed and IFE corrected fluorescence intensity values for S-lansoprazole and R-lansoprazole, respectively. For correction the following equation was used:

$$F_{corr} = F_{obs} \times 10^{\frac{A_{ex}+A_{em}}{2}}$$

where F_{corr} is the IFE-corrected fluorescence; F_{obs} is the observed fluorescence; and A_{ex} and A_{em} are the absorbance values at the excitation and emission wavelengths, respectively.

		S-Lansoprazole		R-Lansoprazole	
	Concentration (μM)	F_{obs}	F_{corr}	F_{obs}	F_{corr}
F_0	0.00	6496	-	6481	-
F_1	1.00	6483	7174	6384	6762
F_2	1.49	6307	6975	6319	6693
F_3	3.45	6020	6814	6104	6611
F_4	5.39	5793	6719	5949	6562
F_5	8.54	5455	6583	5586	6355
F_6	13.74	4926	6376	5429	6482
F_7	22.51	4317	6225	4906	6380
F_8	37.79	2972	5212	4043	6115
F_9	52.92	2426	5093	3199	5620
F_{10}	97.45	1505	5525	2204	6732
F_{11}	140.74	1206	7622	1503	7859

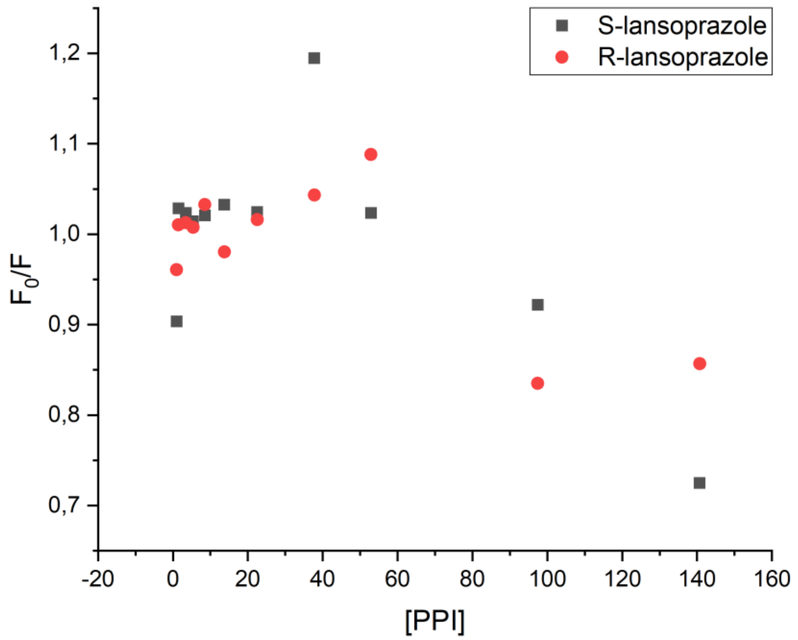

Supplementary Figure 3. Stern-Volmer plots for S-lansoprazole and R-lansoprazole. Although Stern-Volmer plots typically exhibit an increasing trend, the scatter plots here show minimal linearity in the data points ($r^2 = 0.3371$; $r^2 = 0.4126$).

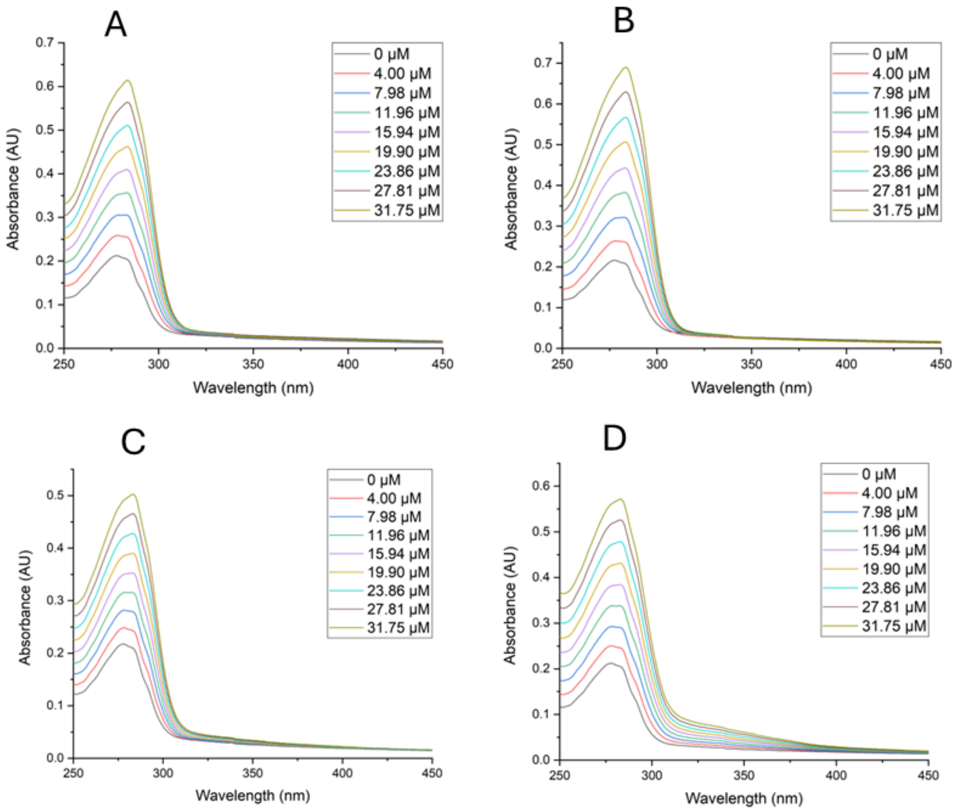

Supplementary Figure 4. Representative UV spectra: A – R-lansoprazole and AGP (1 μM); B – S-lansoprazole and AGP (1 μM); C – R-rabeprazole and AGP (1 μM); D – S-rabeprazole and AGP (1 μM).

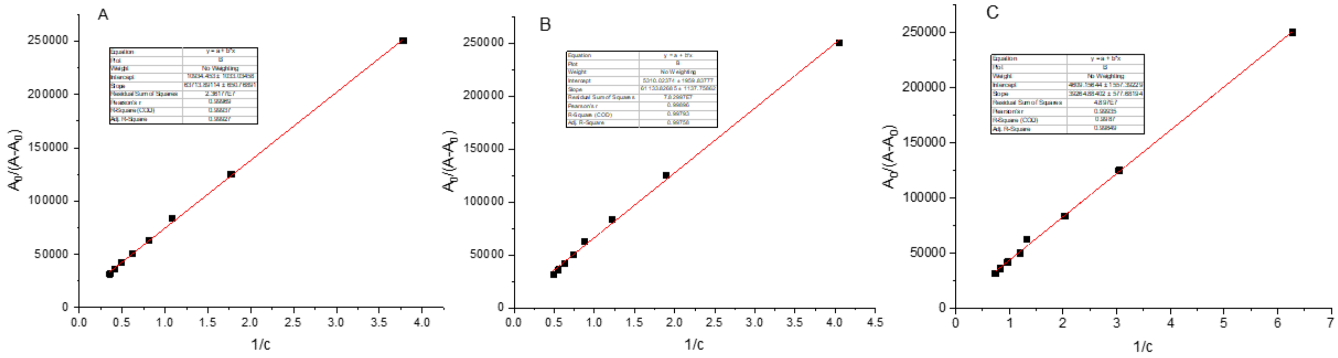

Supplementary Figure 5. Examples of fitting the Benesi-Hildebrandt equation. The r^2 value is higher than 0.995 in all cases: A – S-rabeprazole (r^2 = 0.9994); B – R-omeprazole (r^2 = 0.9979); C – S-omeprazole (r^2 = 0.9979).

Supplementary Table 5. Changes in retention factor values in the presence of warfarin and ibuprofen at different concentrations on HSA column. (Chromatographic parameter: Column temperature: 25 °C, mobile phase: IPA : 10 mM pH 7 sodium phosphate buffer 1:99, flow rate: 0.7 mL/min, detection wavelength: 210 nm).

Site marker	Concentration (mM)	Omeprazole *k*	Rabeprazole *k*	S-lansoprazole *k*	R-lansoprazole *k*
warfarin	0	9.53	7.21	11.17	18.27
	0.01	8.57	5.81	9.03	14.84
	0.05	7.71	4.98	8.13	12.07
	0.1	6.98	4.73	7.44	10.51
ibuprofen	0	9.53	7.21	11.17	18.27
	0.01	7.96	6.67	9.81	13.98
	0.05	6.31	5.87	8.51	10.41
	0.1	5.22	5.05	7.60	7.60

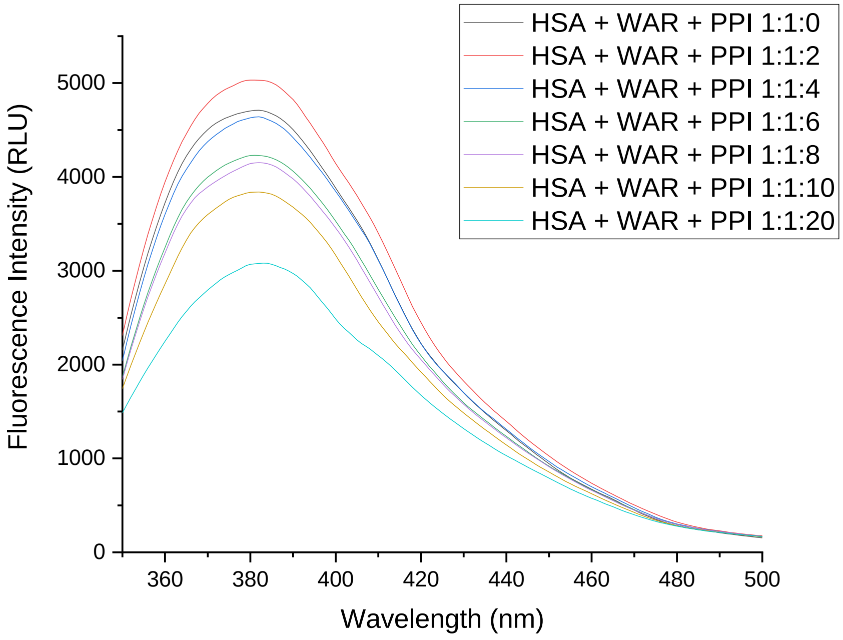

Supplementary Figure 6. The fluorescence spectra of HSA-warfarin complex titrated with S-omeprazole

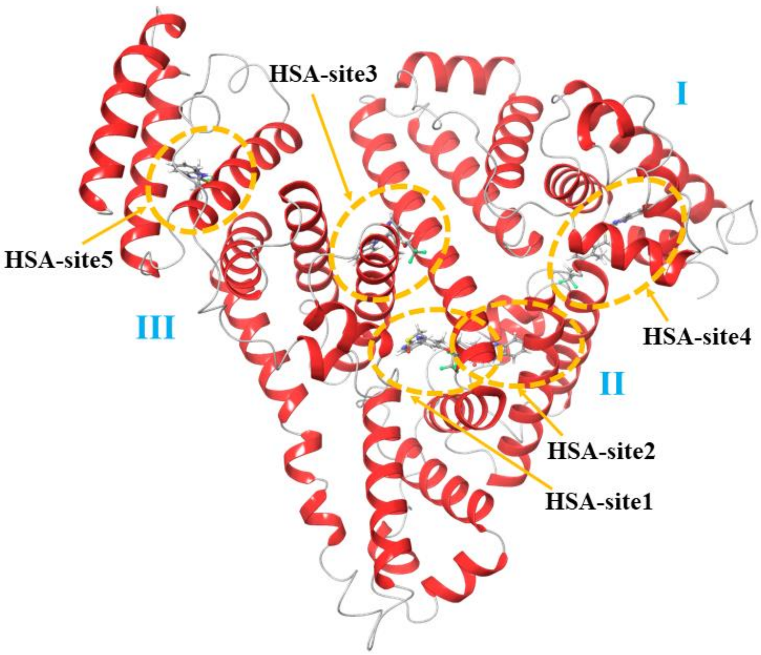

Supplementary Figure 7. The potential binding sites in HSA identified by Sitemap

Supplementary Table 6. Docking scores of PPIs at potential binding sites of HSA

	Docking scores (kcal/mol)					
Binding site (in Suppl. Fig. 7)	R-lansoprazole	S-lansoprazole	R-rabeprazole	S-rabeprazole	R-omeprazole	S-omeprazole
HSA-site1	-5.30	-6.43	-6.50	-6.60	-5.38	-5.09
HSA-site2	-6.67	-7.13	-6.25	-6.56	-5.68	-4.55
HSA-site3	-5.96	-6.18	-6.46	-6.59	-5.36	-5.37
HSA-site4	-8.47	-9.44	-9.78	-10.0	-8.33	-8.19
HSA-site5	-6.03	-7.32	-8.60	-8.45	-6.98	-7.02
